# Type I hair cells of striolar and central zones in vestibular organs are essential for head stability and postural control

**DOI:** 10.1073/pnas.2535179123

**Published:** 2026-06-01

**Authors:** Kazuya Ono, Hyun Jae Lee, Hui Ho Vanessa Chang, Brandie Morris Verdone, Talah Wafa, Youngrae Ji, Austin Huang, Tracy Fitzgerald, Kathleen E. Cullen, Doris K. Wu

**Affiliations:** ^a^ Section on Sensory Cell Regeneration and Development, Laboratory of Molecular Biology, National Institute on Deafness and Other Communication Disorders, National Institutes of Health, Bethesda, MD 20892; ^b^ Department of Biomedical Engineering, School of Medicine, Johns Hopkins University, Baltimore, MD 21205; ^c^ Mouse Auditory Testing Core Facility, National Institute on Deafness and Other Communication Disorders, National Institutes of Health, Bethesda, MD 20892

**Keywords:** vestibular hair cells, vestibular evoked potential, vestibulo-ocular reflex, calyceal synapses, head tremor

## Abstract

The vestibular system of the inner ear provides head motion and orientation information required for maintaining balance and spatial orientation. Each of the five vestibular sensory organs contains type I and type II hair cells (HCs). Type I HCs are particularly notable for their evolutionary adaptability and unique calyceal synapses, in which the vestibular afferent nerve ending envelopes the HC body. In vitro studies indicate that calyceal synapses can transduce signals from HCs to afferents via nonquantal transmission, a mechanism proposed to be faster than conventional bouton synaptic transmission. In specialized regions of vestibular organs—striolae and central zones—many afferents form calyces that encase multiple type I HC bodies, suggesting that nonquantal transmission could be especially important in these regions. Consistently, striolar/central zone afferents are thought to preferentially mediate rapid and high-frequency stimulations. However, the direct consequences of selectively losing these HCs remain unknown. Here, we investigated the role of type I HCs within striolar/central zones by genetically ablating these cells. Reduction of type I HCs in these regions led to a loss of calyces and a compensatory increase in striolar type II HCs. These mutants exhibit reduced vestibular-evoked potentials, a response driven predominantly by striolar activity. In contrast, the vestibulo-ocular reflex, which is thought not to require striolar/central zone function, remained intact. Furthermore, loss of striolar/central zone-specific type I HCs causes head tremor in pups and abnormal head motion in adults, indicating that these HCs are essential for mediating head stability and postural control.

## Significance

Vestibular type I hair cells (HCs) in the inner ear are uniquely enveloped by calyceal nerve endings, a synaptic specialization that emerged in amniotes and is proposed to support head stabilization during the transition from aquatic to terrestrial life. These calyceal synapses enable faster signal transmission than conventional bouton synapses and are concentrated in central zones of vestibular organs. By selectively deleting type I HCs in these regions, we demonstrate that they are required for detecting transient, high-frequency head movements and for maintaining head stability. These findings provide direct functional evidence for type I HC–calyx complexes in central zones of vestibular organs and advance understanding of how vestibular organs encode head motion.

**Fig. 1.**
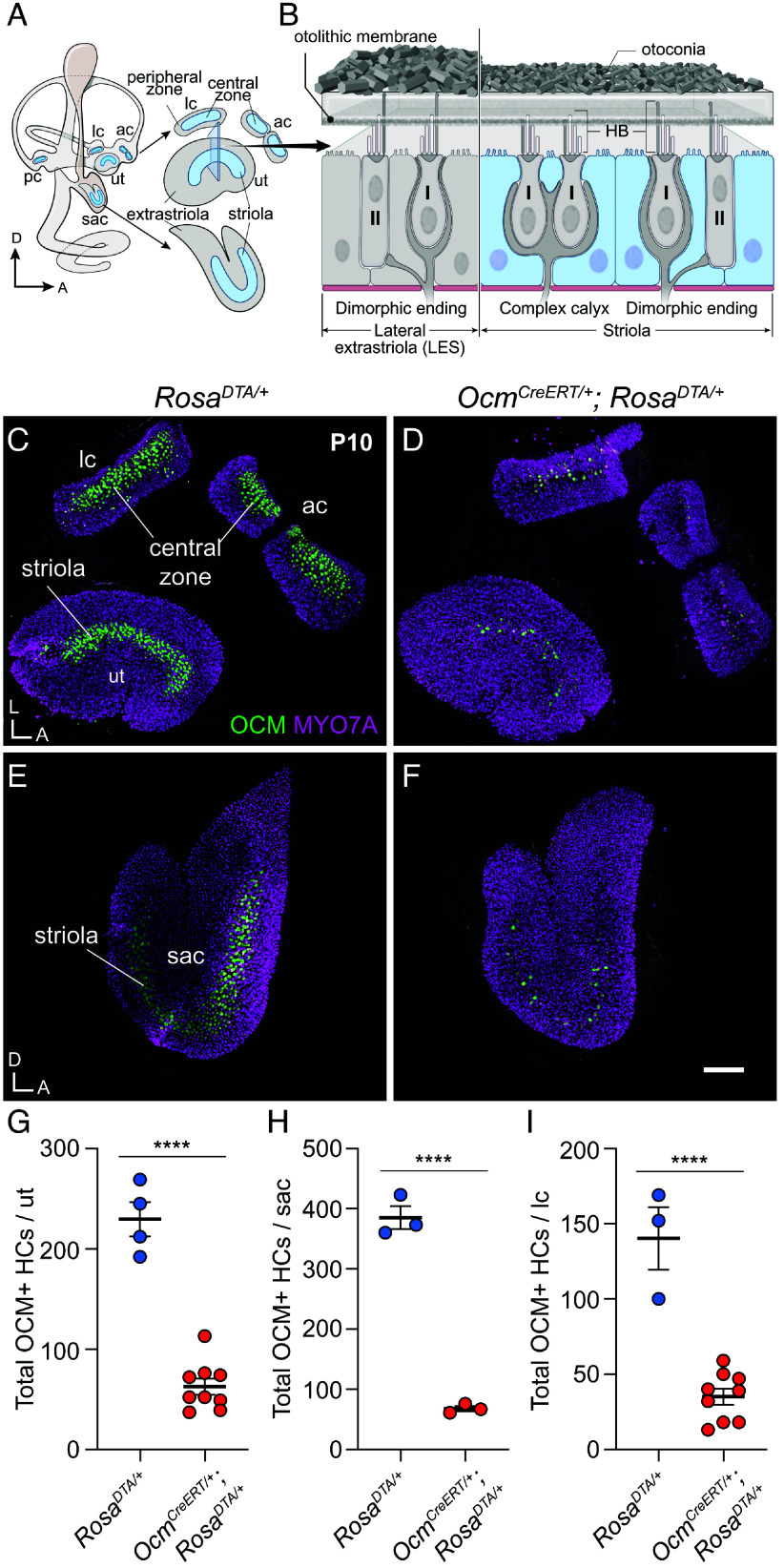
Ablation of OCM^+^ type I HCs of the striola/central zone in *Ocm^CreERT/+^; Rosa^DTA/+^* mice. (*A*) Schematic diagram of the mouse inner ear highlighting the five vestibular sensory organs (ac, lc, pc, sac, and ut) and their zones. The central zone of the anterior and lateral crista (ac, lc) and the striola of the utricle (ut) and saccule (sac) are shown in blue. The peripheral zone of the cristae and extrastriola of the utricle and saccule are shown in gray. (*B*) A cross-sectional view of the lateral extrastriola (LES) and striola of the mouse utricle from (*A*), showing the anatomical differences between the two zones, including thinner otoconia sitting above the hair bundles (HB) and the presence of pure calyces in the striola. (*C*–*F*) P10 whole-mount inner ears of *Rosa^DTA/+^* (*C* and *E*) and *Ocm^CreERT/+^; Rosa^DTA/+^* mice (*D* and *F*) after tamoxifen administration at P0 and P1, and immunolabeled with anti-oncomodulin (OCM, green) and anti-myosin7a (MYO7A, magenta) antibodies. OCM is expressed in the striola of utricle (ut) and saccule (sac) and central zones of anterior and lateral cristae (ac, lc). (*G*–*I*) Quantification of total OCM+ HCs in utricles (*G*, 229.5 ± 17.10, n = 4), saccule (*H*, 385.3 ± 19.20, n = 3), and lateral crista (*I*, 140.3 ± 20.75, n = 3) of *Rosa^DTA/+^* controls (blue). Compared to controls, OCM+ HCs are reduced in utricles (*G*, 62.7 ± 7.95, n = 9, 72.7% reduction, *P* < 0.0001), saccules (*H*, 68.0 ± 4.35, n = 3, 82.4% reduction, *P* < 0.0001), and lateral cristae (*I*, 35.1 ± 5.34, n = 9, 75.0% reduction, *P* < 0.0001) of *Ocm^CreERT^*^/+^; *Rosa^DTA/+^* mutants (red). Error bars: SEM. A, anterior; L, lateral; D, dorsal. The Scale bar in F equals 200 µm and applies to *C*–*E*.

Approximately 10,000 sensory hair cells (HCs) are distributed among the five vestibular organs of the mouse inner ear—three cristae and two otolith organs—which detect angular and linear head motion, respectively (Fig. 1*A*). Each organ contains two types of HCs (Fig. 1 *B*, *I* and *II*). Type I HCs are particularly notable for their unique calyceal synapses, in which nerve endings of vestibular afferents encase HC bodies, whereas afferents only form conventional bouton synapses with type II HCs (Fig. 1*B*).

The close apposition between HC and calyceal membranes enables electrical coupling between HCs and afferents known as nonquantal transmission, which provides greater speed and sensitivity over conventional bouton synapses (1–3). This advantage is thought to be enhanced in the striola of otolith organs and central zone of cristae (Fig. 1*A*), where vestibular afferents can encase single or multiple type I HC bodies (up to three). In contrast to these pure and complex calyces, most afferents present in the organ are dimorphic, forming both calyces and bouton synapses on type I and type II HCs, respectively (4).

Type I HCs and their calyceal nerve endings are unique to amniotes and are thought to have evolved to meet the increased demands of head motion controls as animals transition from aquatic to land environment. In mammals, unlike other amniotes, type I HCs are not restricted to the striolar/central zones but are broadly distributed across the vestibular organs (5, 6). Despite this evolutionary adaptability, the precise functional contributions of type I HCs and how these contributions differ across zones, remain unclear.

Vestibular afferents also exhibit zonal differences. Afferents innervating the striolar/central zones exhibit variable, irregular resting discharge rates (irregular afferents), whereas those innervating the surrounding extrastriolar/peripheral zones display more regular discharge patterns (regular afferents). Current models propose that these two afferent classes employ different sensory decoding strategies: Irregular afferents are specialized for high-frequency, transient stimuli, whereas regular afferents are tuned for low-frequency, sustained stimuli (4, 7). This dogma is largely based on physiological and computational studies that classified afferents based on their resting discharge properties. Although the firing patterns of irregular and regular afferents are thought to reflect inputs from respective type I and type II HCs (1, 2), it is unclear whether the central functions attributed to irregular afferents (7, 8) depend specifically on type I HCs in the striolar/central zones.

In addition to zonal differences in afferent discharge pattern, type I HCs and their afferents exhibit different molecular (5, 6, 9–11) and physiological properties (4) across zones. For example, only type I HCs in striolar/central zones express a calcium binding protein, OCM (12). To investigate the specific contribution of type I HCs in the striolar/central zones, distinct from those in extrastriolar/peripheral zones, we selectively deleted these cells using diphtheria toxin driven by an inducible *cre* under the *Ocm* promoter. Postnatal depletion of type I HCs resulted in secondary loss of vestibular afferents but a compensatory increase in type II HCs. Functionally, mutants exhibited reduced vestibular evoked potentials (VsEPs), which are elicited by fast, jerk stimuli to the otolith organs (13), whereas the vestibulo-ocular reflex (VOR) which maintains gaze, remained normal. Behaviorally, mutants showed postnatal head tremor and instability on the balance beam in adulthood. Together, these findings indicate that type I HCs in striolar/central zones are required for detecting transient, high-frequency stimuli to ensure the maintenance of postural stability.

## Results

### *Ocm^CreERT^* Strain Targets Striolar/Central Zone HCs.

To investigate the functions of type I HCs in striolar/central zones, we generated an *Ocm^CreERT^* mouse strain, in which *CreERT* was knocked into the *Oncomodulin* (*Ocm*) locus. *Ocm* encodes a calcium binding protein expressed in the mouse inner ear but not in the brain during development (*SI Appendix*, Fig. S1), and OCM is enriched in type I HCs of striolar/central zones (12). To assess Cre activity, we crossed *Ocm^CreERT^* mice with *Rosa^tdTomato^* reporter and induced recombination with tamoxifen administration at P0 (postnatal day 0) and P1. By P3, tdTomato immunoreactivity (tdTomato+) overlapped with the majority of OCM+ HCs across vestibular organs, including utricles, saccules, and cristae (*SI Appendix*, Fig. S2). A small number of tdTomato+ but OCM- HCs likely represent low OCM immunoreactivity below detection and/or Cre leakiness. Overall, OCM+ and tdTomato+ HC numbers were comparable (*SI Appendix*, Fig. S2 *C*–*E*), and reporter activity was not detected outside the inner ear. These results indicate that the *Ocm^CreERT^* strain largely recapitulates endogenous OCM expression and enables selective targeting of striolar/central zone type I HCs.

### Selective Ablation of Striolar/Central Zone Type I HCs.

To selectively ablate type I HCs in striolar/central zones, we crossed the *Ocm^CreERT^* mice with *Rosa^DTA/DTA^*, in which the diphtheria toxin gene is knocked into the *Rosa* locus. *Ocm^CreERT/+^; Rosa^DTA/+^* double heterozygous mice were injected with tamoxifen at P0 and P1 and harvested 9 d later, the approximate time period required for diphtheria toxin-mediated HC ablation (14). Tamoxifen treatments did not affect the overall growth or survival of the mutants. Compared with *Rosa^DTA/+^* controls (Fig. 1 *C, E,* and *G–I*), tamoxifen-treated *Ocm^CreERT/+^; Rosa^DTA/+^* mutants exhibited a greater than 70% reduction in OCM+ HCs in vestibular organs by P10 (Fig. 1 *D* and *F–I*), indicating effective ablation.

**Fig. 2.**
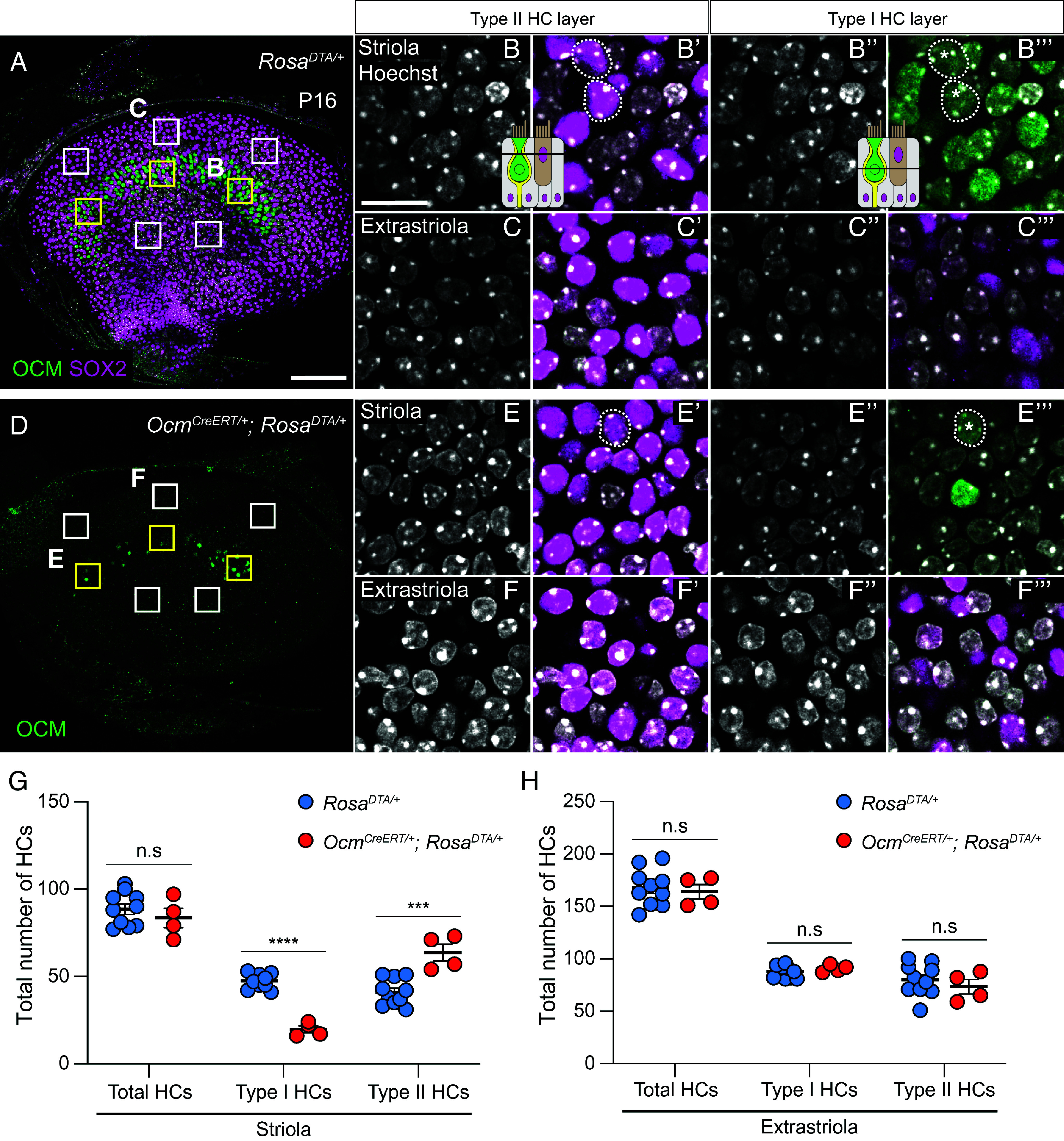
Increased SOX2+ type II HCs in *Ocm^CreERT/+^; Rosa^DTA/+^* mice. (*A*–*F*″′) P16 whole-mount utricles from control (*A*–*C*″′) and mutant (*D*–*F*″’) mice immunolabeled with anti-OCM (green) and anti-SOX2 (magenta) antibodies. A single plane taken from z-stack confocal images of a control (*A*) and mutant (*D*) utricle. Panels (*B*–*C*″′) and (*E*–*F*″′) show enlarged views of the selected areas from control and mutant utricles, respectively. The line in the schematic of (*B* and *B*′) indicates the level of optical section at the level of type II HC nuclei and it applies to panels (*C*, *C*′, *E*, *E*′, *F*, and *F*′). The line in the schematic of (*B*″and *B*″′) indicates the optical section at the level of type I HC nuclei and it applies to panels (*C*″, *C*″’, *E*″, *E*″′, *F*″, and *F*″′). Type II HC nuclei that are positive for both SOX2 and OCM are outlined with a white dotted line and marked with an asterisk. (*G* and *H*) Quantification of HCs from the three striolar regions (*G*, yellow squares in *A* and *D*) and the five extrastriolar regions (*H*, white squares in *A* and *D*, including three in lateral and two in medial extrastriolar regions; 44 × 44 µm each). (*G*) In the striolar region, total HC numbers were not significantly different between controls (blue) and mutants (red) (88.6 ± 3.02, n = 10 controls vs. 83.5 ± 5.56, n = 4 mutants; *P =* 0.40). However, striolar type I HCs in mutants were reduced to 58.4% of controls (47.5 ± 1.32, n = 10 controls vs. 19.7 ± 1.93, n = 4 mutants; *P* < 0.000001), whereas striolar type II HCs were increased by 55.1% (41.1 ± 2.38, n = 10 controls vs. 63.7 ± 4.82, n = 4 mutants; *P =* 0.000497). (*H*) In the extrastriola, there were no differences between control (blue) and mutant (red) utricles in the number of total HCs (167.9 ± 5.54, n = 10 controls vs. 164.3 ± 6.82, n = 4 mutants; *P =* 0.72), type I HCs (87.8 ± 1.84, n = 10 vs. 90.7 ± 1.75, n = 4; *P =* 0.37), or type II HCs (80.1 ± 4.81, n = 10 vs. 73.5 ± 6.86, n = 4; *P =* 0.47). The scale bar in *A* equals 200 µm and applies to *D*; the scale bar in *B* equals 50 µm and applies to *B*′ to *F*″′.

To confirm loss of type I HCs independent of OCM staining, we quantified striolar type I HCs in the utricle by subtracting Sox2+ type II HCs (15), from the total number of Hoechst+ HCs. We analyzed samples at P16 rather than P10 because approximately 50% of HCs in the mouse utricle are generated postnatally (16) and total HC number in the mouse utricle reaches maturity by around P12 (17). Within the sensory epithelium, type I HC nuclei are positioned more basally than type II nuclei [Figs. 1 and 2, schematics; (15)]. Using this method, we found no change in total HC number in the striola of mutants compared to *Rosa^DTA/+^* controls. However, there was a 58.4% reduction in type I HCs in the striola of mutants (Fig. 2 *A, B–B″′, D, E–E″′,* and *G*, yellow squares). This reduction is largely consistent with the 73% decrease in OCM+ HCs observed at P10 (Fig. 1*G*), indicating that OCM immunoreactivity is a good readout of striolar HCs in this deletion mutant. No reduction was observed in the five selected extrastriolar regions (Fig. 2 *A, C–C″′, D, F–F″′,* and *H*, white squares). Together, these results indicate a specific loss of striolar/central zone type I HCs in *Ocm^CreERT/+^; Rosa^DTA/+^* mutants.

### Compensatory Increase of Type II HCs in *Ocm^CreERT/+^; Rosa^DTA/+^* Mutants.

Despite loss of type I HCs, total HC number in the mutant striola remained constant, suggesting a compensatory increase in type II HCs. Indeed, the total number of SOX2+ type II HCs increased by 55.1% in the mutant striola, compared to controls (Fig. 2 *A, B, B′, D, E, E′,* and *G*, yellow squares), with no change in extrastriolar regions (Fig. 2 *C*, *C′, F, F′,* and *H*, white squares).

In the adult mouse utricle, approximately 9.8% of striolar type II HCs are also OCM+ (18). To determine whether OCM+ type II HCs were changed in *Ocm^CreERT/+^; Rosa^DTA/+^* mutants, we analyzed samples that were coimmunostained for OCM and SOX2. In control P16 utricles, 9.0% of striolar type II HCs were OCM+ (Fig. 2 *B*–*B″*′**, white dotted outline with asterisk; n = 10 utricles, three yellow squares per utricle), consistent with published results (18). In *Ocm^CreERT/+^; Rosa^DTA/+^* mutants, the total number of OCM+ type II HCs was not significantly different from controls (Fig. 2 *B*–*B″*′**and *E–E″*′**, white dotted outline with asterisk; control: 3.7 ± 1.07, n = 10; mutant: 1.75 ± 1.03, n = 4 utricles). However, the proportion of OCM+ type II HCs decreased to 2.75%, reflecting a compensatory increase in SOX2-only type II HCs. Collectively, these results indicate that neonatal deletion of OCM+ HCs results in a substantial loss of striolar type I HCs, accompanied by a region-specific compensatory increase in SOX2-only type II HCs.

### Otoconia Clearance Remains Intact in *Ocm^CreERT/+^; Rosa^DTA/+^* Mutants.

The otoconia is an extracellular matrix, which sits on top of the sensory epithelium of the utricle and saccule, where hair bundles of HCs are embedded (Fig. 1*B*). The otoconia above the striola of the mouse utricle is thinner than in the surrounding regions, giving the striola a translucent appearance and thus marking its location [Fig. 1*B* and *SI Appendix*, Fig. S3, (19)]. In mutants with selective type I HC deletion at P8 and P16, otoconia clearance within the striola was preserved and resembled that of *Rosa^DTA/+^* controls (*SI Appendix*, Fig. S3). The preservation of otoconia clearance indicates that the HCs and/or supporting cells responsible for this specialization (20, 21) are not affected by selective type I HC deletion.

### Reduction of Striolar Calyces After HC Ablation.

**Fig. 3.**
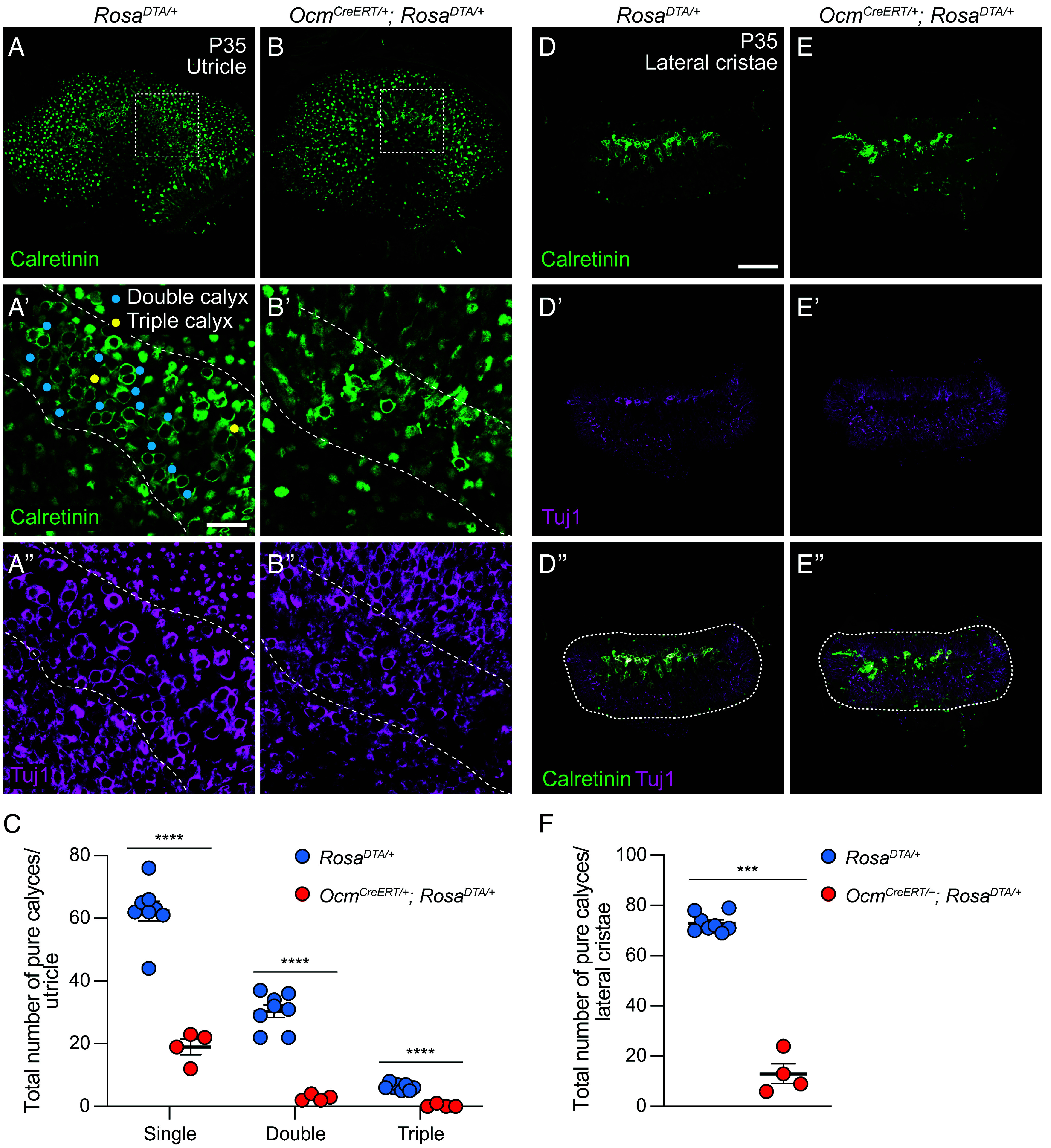
Loss of calretinin-positive afferent terminals in *Ocm^CreERT/+^; Rosa^DTA/+^* utricles and lateral crista. Single-plane images (*A′,A*″, *B′,* and *B*″) selected from confocal Z-stack images of whole-mount control (*A*) and *Ocm^CreERT/+^; Rosa^DTA/+^* (*B*) mutant utricles at P35. Mice were injected with tamoxifen at P0 and P1. Utricles were immunolabeled with anti-calretinin (green) and anti-Tuj1 (magenta) antibodies. In both control and mutant utricles, calretinin labels striolar pure calyces (halo-shaped) and type II HCs (solid staining). (*A′* and *A*″) Inset of (*A*) showing higher magnification view of the striolar region (marked with dotted lines) reveals calretinin-positive (*A′*) and Tuj1-positive (*A*″, neuronal marker) pure/complex calyces that encased single, double (blue dots), or triple (yellow dots) HCs, which are markedly reduced in mutants (*B′* and *B*″). (*C*) Quantification of pure/complex calyces in control and mutant utricles. In the mutant striola (*Inset* in *B*), single calyces were reduced by 69.5% (62.4 ± 3.12, n = 8 controls vs.19.00 ± 2.48, n = 4 mutants; *P* < 0.000001). Double calyces were reduced by 90.9% (30.4 ± 2.04, n = 8 controls vs. 2.8 ± 0.48, n = 4 mutants; *P =* 0.000001). Triple calyces were reduced by 96.0% (6.3 ± 0.37, n = 8 controls vs. 0.3 ± 0.25, n = 4 mutants; *P* < 0.000001). The total number of pure calyces per utricle was reduced by 77.8% (control 99 ± 4.90 vs. mutant 22 ± 2.86). (*D*–*D*″ and *E*–*E*″) Images of a lateral crista from control (*D*–*D*″) and mutant (*E*–*E*″) stained with anti-calretinin (*D* and *E*) and anti-Tuj1 (*D*′ and *E*′), showing calretinin-positive pure/complex calyces in the central zone of control (*D*–*D*″) are reduced in the mutant (*E*–*E*″). (*F*) Quantification of pure calyces in control (blue) and mutant (red) lateral cristae. Pure calyces are reduced by 82.2% in mutants compared to controls (73 ± 1.31, n = 8 in controls vs 13 ± 3.94, n = 4 in mutants, *P* = 0.000223). [Scale bar, 200 µm (*A* and *D*), 50 µm (*A*′).]

The loss of sensory HCs causes degeneration of afferent fibers in the auditory system (22, 23). We next examined the effects of diphtheria toxin-mediated HC death on neuronal innervation. *Ocm^CreERT/+^; Rosa^DTA/+^* utricles treated with tamoxifen at P0 and P1 were harvested in adulthood (P35) and immunolabeled with antibodies for Tuj1, a pan-neuronal marker, and calretinin, a Ca^2+^ binding protein expressed only in pure calyceal nerve endings of striolar/central zones after mice reach maturity (5, 6, 24). Both anti-Tuj1 and anti-calretinin staining were reduced in *Ocm^CreERT/+^; Rosa^DTA/+^* mutant utricles (Fig. 3 *A*–*C*) and lateral cristae (Fig. 3 *D*–*F*), compared to *Rosa^DTA/+^* controls. The number of complex calyces within the striola serves as an index of complexity (6). The *Rosa^DTA/+^* controls showed a distribution of 6:3:1 ratio of single, double, and triple calyces in the mouse striola of the utricle (Fig. 3*C*), consistent with previous reports (6). Compared to *Rosa^DTA/+^* controls, we observed a marked reduction across all pure calyces, with an overall 77.8% decrease in the utricle (Fig. 3*C*) and an 82.2% reduction in the lateral crista (Fig. 3*F*) of *Ocm^CreERT/+^; Rosa^DTA/+^* mutants. Together, these results indicate that diphtheria toxin-mediated type I HC loss causes a secondary loss in calyceal nerve endings.

### Normal VOR After Depletion of Striolar/Central Zone Type I HCs.

We next assessed the functional consequences from postnatal loss of striolar/central zone type I HCs and their calyceal nerve endings in *Ocm^CreERT/+^; Rosa^DTA/+^* mutant mice. VOR response to low frequency stimulations, which is thought not to require striolar/central zone input (25), was normal in the *Ocm^CreERT/+^; Rosa^DTA/+^* mutants (*Materials and Methods* and *SI Appendix*, Fig. S4 and Tables S1–S3). The optokinetic reflex (OKR) was also normal in the mutants. These results indicate that visual-vestibular integration for gaze stabilization remains intact despite the reduction of striolar/central zone type I HCs.

### Reduced VsEPs After Depletion of Striolar/Central Zone Type I HCs.

**Fig. 4.**
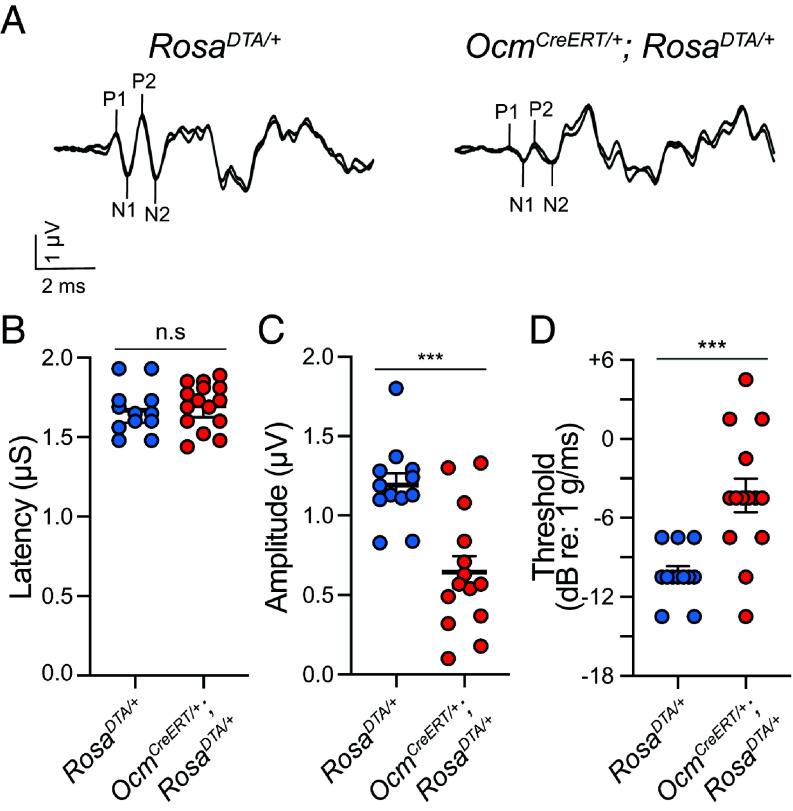
Linear VsEPs are affected in *Ocm^CreERT/+^; Rosa^DTA/+^* mice. (*A*) Two representative VsEP waveforms recorded at maximal jerk level (+6 dB) from *Rosa^DTA/+^* controls (*Left*) and *Ocm^CreERT/+^; Rosa^DTA/+^* mutants (*Right*). The VsEP waveform in the *Ocm^CreERT/+^; Rosa^DTA/+^* mutant shows a reduced P1-N1 amplitude. (*B*–*D*) Quantification of P1-N1 latency (*B*), response amplitude (*C*), and threshold stimulus levels (*D*). In *Rosa^DTA/+^* controls (blue), responses exhibit a series of discernable positive (P1, P2) and negative peaks (N1, N2) with latencies of approximately 2 ms (1.67 ± 0.04 ms, n = 12) and P1-N1 amplitudes of 1.19 ± 0.07 µV (n = 12). In *Ocm^CreERT/+^; Rosa^DTA/+^* mutants (red), latencies were not significantly different from controls (1.70 ± 0.04 ms, n = 15, *P =* 0.6179) but P1-N1 amplitudes were significantly reduced (0.65 ± 0.10 µV, n = 14 vs. 1.19 ± 0.07, 45% reduction, *P =* 0.0003). Response thresholds were elevated in *Ocm^CreERT/+^; Rosa^DTA/+^* mutants (−4.29 ± 1.28 dB, n = 14, 58.2% reduction, *P =* 0.0005) compared to controls (−10.25 ± 0.58 dB, n = 12).

VsEPs, elicited in anesthetized mice by repeatedly applying transient linear jerk stimuli to the head, are postulated to be a readout of striolar function in the utricle and saccule (13, 26, 27). We measured VsEPs in *Ocm^CreERT/+^; Rosa^DTA/+^* adult mutants after postnatal administration of tamoxifen, and we found that *Rosa^DTA/+^* controls exhibited typical VsEP response waveforms, showing a P1 latency of 1.67 ms and average P1-N1 amplitude of 1.19 µV (Fig. 4 *A*–*C*). In *Ocm^CreERT/+^; Rosa^DTA/+^* mutants, VsEPs response latencies (P1, N1) were unchanged. However, response amplitudes (P1-N1) at maximum stimulus were significantly reduced (Fig. 4 *A*–*C*, avg 0.65 µV, a 45.9% reduction compared to controls). In addition, the stimulus thresholds required to elicit a minimal P1-N1 response were elevated in *Ocm^CreERT/+^; Rosa^DTA/+^* mutants (avg −4.29 dB re: 1 g/ms), compared to *Rosa^DTA/+^* controls (Fig. 4*D*, −10.25 dB re: 1 g/ms). Taken together, these results indicate that VsEPs are compromised by the depletion of striolar type I HCs, supporting the hypothesis that VsEPs are generated by the striolae of the utricle and saccule.

### Head Tremor in *Ocm^CreERT/+^; *Rosa*^DTA/+^* Pups.

We observed pronounced head tremor in *Ocm^CreERT/+^; Rosa^DTA/+^* pups at P8 after tamoxifen administration at birth. While the heads of control pups tend to oscillate during movements as well, the oscillations were more prominent and sustained in mutants (Movie S1). To better quantify the head tremor phenotype, we affixed an inertial measurement unit (IMU) to the heads of pups and observed them in an open arena.

**Fig. 5.**
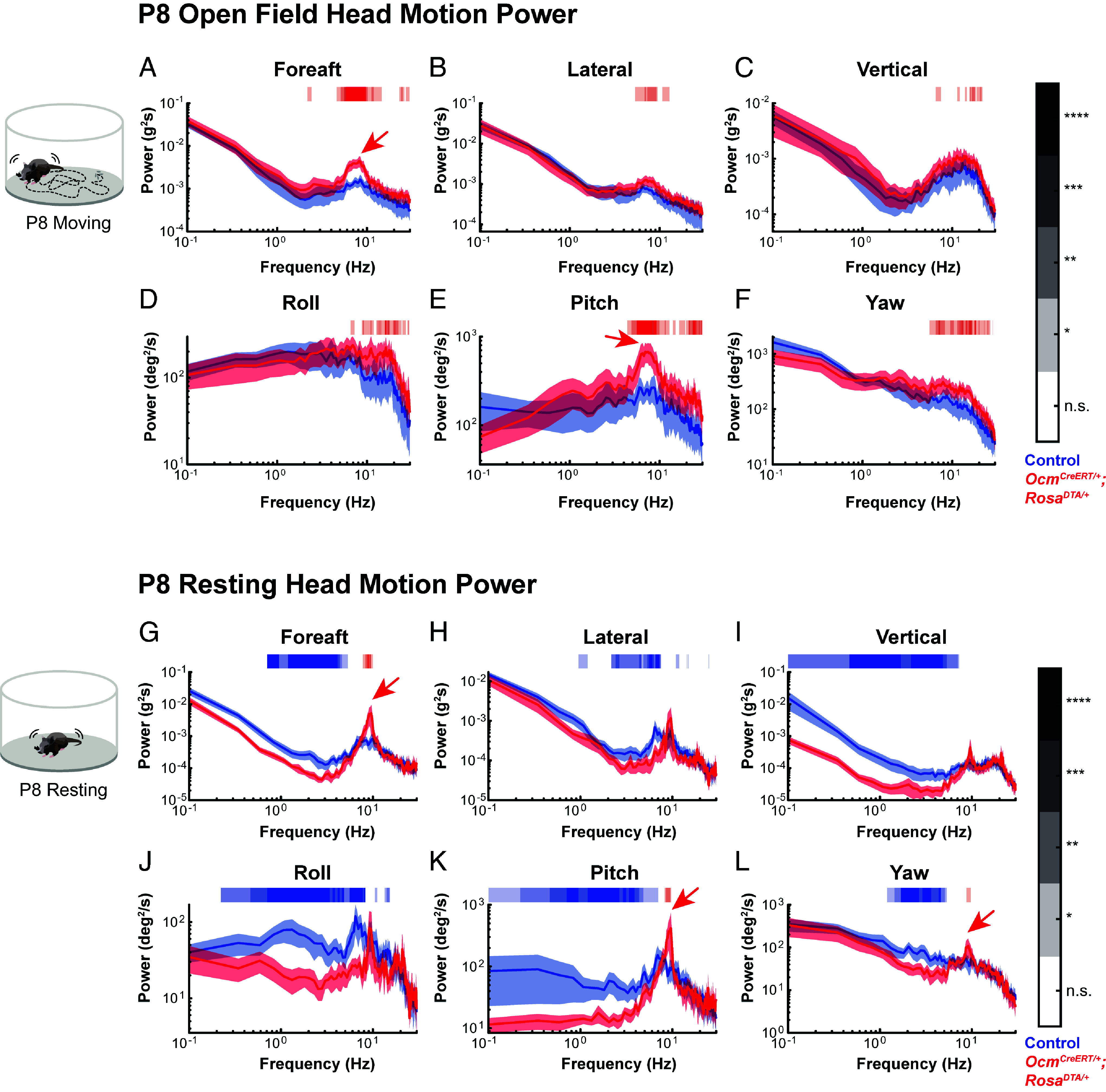
*Ocm^CreERT/+^; Rosa^DTA/+^* pups demonstrate dynamic postural differences when traversing an open arena and at rest. (*A*–*F*) P8 head motion power spectra in the translational acceleration (*A*–*C*) and rotational velocity (*D*–*F*) domains during an open field task. n = 18 control (blue) and 10 *Ocm^CreERT/+^; Rosa^DTA/+^* (red) mice. Bars indicate significance value at that frequency window colored according to the group with the higher motion power. Arrows indicate a significant peak in power for *Ocm^CreERT/+^; Rosa^DTA/+^* mice at approximately 7 to 9 Hz in the foreaft and pitch domains, compared to controls. (*G*–*L*) P8 head motion power spectra in the translational acceleration (*G*–*I*) and rotational velocity (*J*–*L*) domains during rest periods in an open field between controls (blue, n = 18) and *Ocm^CreERT/+^; Rosa^DTA/+^* (red, n = 10) mice. Bars indicate significance value at that frequency window colored according to the group with the higher motion power. Arrows indicate a significant peak in power of *Ocm^CreERT/+^; Rosa^DTA/+^* mice at approximately 9 Hz in the foreaft, pitch, and yaw domains, compared to controls. Mean ± shaded SEM.

Motion power during periods of movement and rest in both mutants and littermate controls were observed and quantified across six dimensions of freedom. During movements, elevated motion power at higher frequencies was observed across all six axes in both *Ocm^CreERT/+^; Rosa^DTA/+^* mutant and littermate *Rosa^DTA/+^* control pups at P8 (Fig. 5 *A*–*F*). However, a significant peak in the 7 to 10 Hz range was readily observed in mutants, specifically in foreaft acceleration and pitch velocity (Fig. 5 *A* and *E*, red arrows). These results are consistent with more pronounced oscillatory head movements in mutants compared to controls (Movie S1). Notably, this high-power frequency signature persisted in *Ocm^CreERT/+^; Rosa^DTA/+^* pups during periods of rest (Fig. 5 *G*–*L*, red traces) in otherwise hypoactive head movements of lower frequencies compared to controls (blue traces). This effect was most pronounced in foreaft acceleration, and rotations in pitch and yaw (Fig. 5 *G, K,* and *L*, red arrows). Together, these data indicate that type I HCs of striolar/central zones play a key role in baseline head stabilization, both at rest and during active movement.

**Fig. 6.**
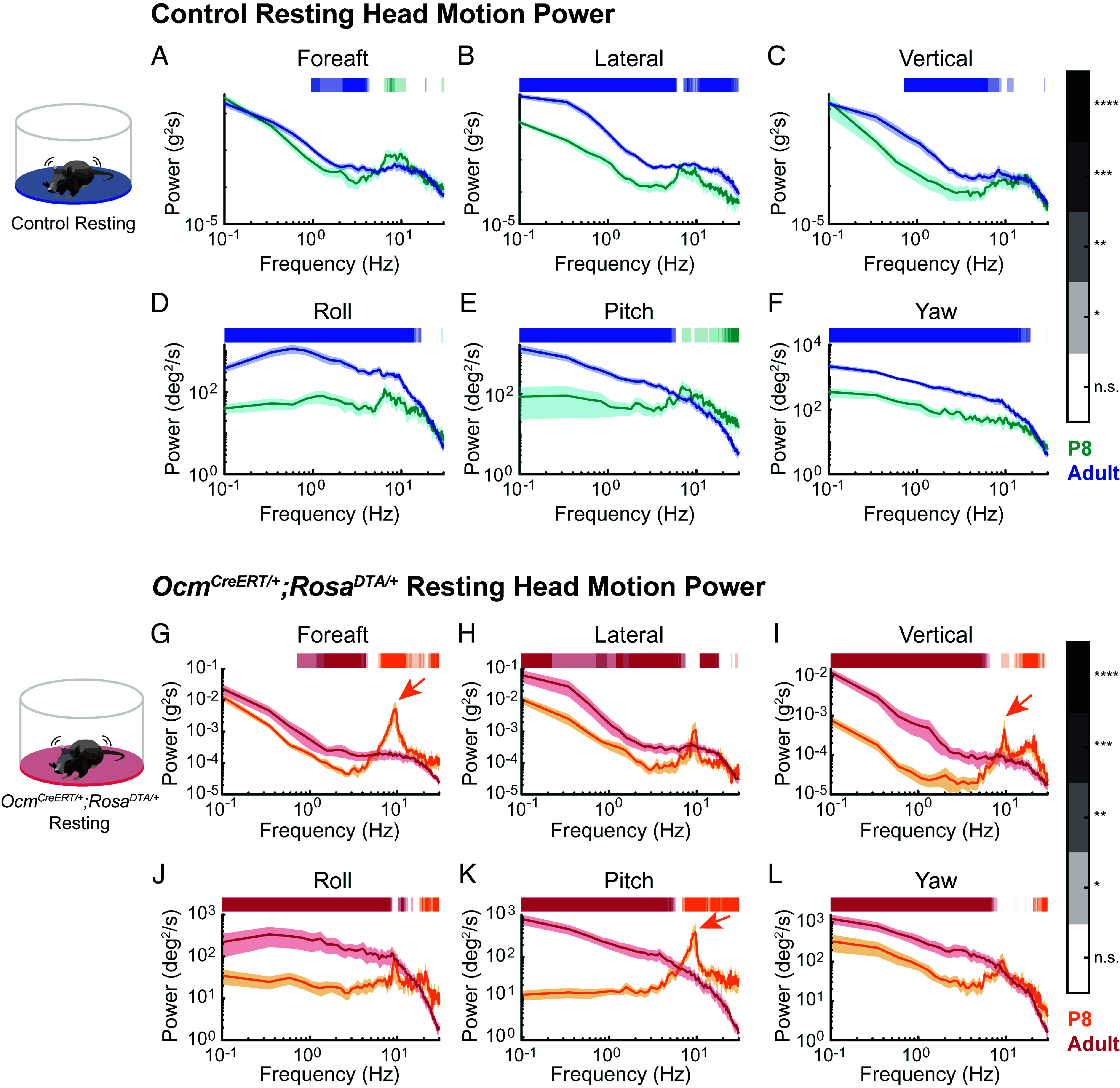
Age-dependent changes in translational and rotational axes at rest in controls and *Ocm^CreERT/+^; Rosa^DTA/+^* mutants. Comparisons between P8 (light blue, data taken from *G*–*L*; n = 18) and adult (blue, n = 8) *Rosa^DTA/+^* control mice in head motion power spectra in the translational acceleration (*A*–*C*) and rotational velocity (*D*–*F*) domains during rest periods in the open field task. Bars indicate significance value at that frequency window colored according to the group with the higher motion power. (*G*–*L*) Comparisons between P8 (orange, data taken from *G*–*L*; n = 10) and adult *Ocm^CreERT/+^; Rosa^DTA/+^* mice (red, n = 8) in head motion power spectra in the translational acceleration (*G*–*I*) and rotational velocity (*J*–*L*) domains during rest periods in the open field task. Bars indicate significance value at that frequency window colored according to the group with the higher motion power. Arrows indicate a significant peak in power spectrum of P8 *Ocm^CreERT/+^; Rosa^DTA/+^* mice at approximately 9 Hz in the foreaft, vertical, and pitch domains, compared to adults. Mean ± shaded SEM.

As *Rosa^DTA/+^* control mice mature, the elevated motion power at higher frequencies observed at P8 (Fig. 6 *A*–*F*, light blue) were no longer detectable in the adult, and there was an overall increase in head motion power across frequencies during rest (Fig. 6 *A*–*F*, blue). A similar generalized increase in motion power across frequencies was observed in adult *Ocm^CreERT/+^; Rosa^DTA/+^* mice (Fig. 6 *G*–*L*, red) compared to mutant pups (orange), and the distinct 7 to 10 Hz tremor peaks and high-frequency power elevation observed at P8 did not persist into adulthood (Fig. 6 *G*–*L*, orange arrows). These results are consistent with the observation that the overt head tremor observed in mutant pups was no longer visually apparent in adults.

**Fig. 7.**
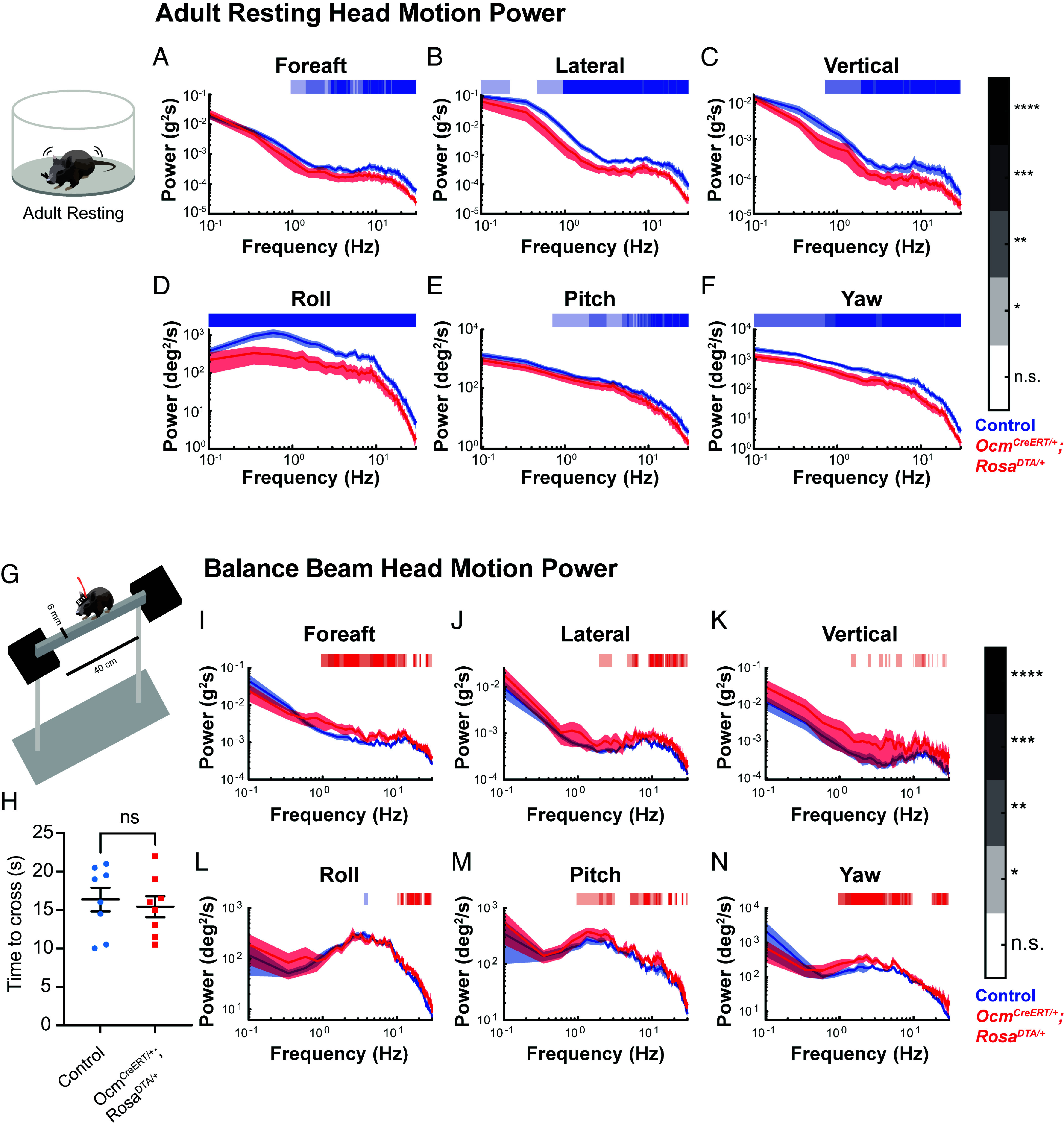
Adult *Ocm^CreERT/+^; Rosa^DTA/+^* mice demonstrate dynamic postural differences at rest and while traversing a narrow balance beam. Adult head motion power spectra in the translational acceleration (*A*–*C*) and rotational velocity (*D*–*F*) domains during rest periods of an open field task of adult (blue, data taken from Fig. 6 *A*–*F*; n = 8) and *Ocm^CreERT/+^; Rosa^DTA/+^* mice (red, data taken from Fig. 6 *G*–*L*; n = 8). Bars indicate significance value at that frequency window colored according to the group with the higher motion power. (*G*) Schematic of a mouse with an IMU affixed to its head, traversing a 40 cm long balance beam of 6 mm diameter. (*H*) Quantification of crossing time for control (blue, n = 8) and *Ocm^CreERT/+^; Rosa^DTA/+^* (red, n = 8) mice. Time for controls and *Ocm^CreERT/+^; Rosa^DTA/+^* mice to cross the balance beam are 16.4 ± 1.56 s and 15.4 ± 1.36 s, respectively. *P =* 0.7402, unpaired, two-tailed *t* test. (*I*–*N*) Head motion power spectra in the translational acceleration (*I*–*K*) and rotational velocity (*L*–*N*) domains of control (blue, n = 8) and *Ocm^CreERT/+^; Rosa^DTA/+^* (red, n = 8) mice. Bars indicate significance value at that frequency window colored according to the group with the higher motion power. Mean ± shaded SEM.

Compared to adult controls at rest (Fig. 7 *A*–*F*, blue), *Ocm^CreERT/+^; Rosa^DTA/+^* mutants showed a global reduction in head motion power, particularly at frequencies above 1 Hz (red). Together, these findings indicate that early postnatal head instability largely is resolved by adulthood, with only subtle, consistent reduction in motion power at higher frequencies.

### Dynamic Postural Differences in Mutant Mice Compared to Controls.

To assess the functional significance of head instability in adult *Ocm^CreERT/+^; Rosa^DTA/+^* mice, we next tested their ability to traverse a narrow (6 mm) elevated balance beam while wearing an IMU (Fig. 7*G*). The time required to cross the narrow beam did not differ between *Ocm^CreERT/+^; Rosa^DTA/+^* (red) and *Rosa^DTA/+^* control (blue) mice (Fig. 7*H*), suggesting that basic locomotor coordination and balance under this challenging condition were preserved in the mutants. However, despite the normal beam traversing-time, *Ocm^CreERT/+^; Rosa^DTA/+^* mice displayed significantly elevated head motion power, particularly at higher frequencies above 1 Hz, while traversing the beam (Fig. 7 *I*–*N*, red traces). Thus, although gross locomotor coordination and balance were maintained, the increased high-frequency head motion during the narrow beam task reveals underlying vestibular deficits in the mutants.

## Discussion

### Cellular Consequences from Depletion of Striolar/Central Zone Type I HCs.

In *Ocm^CreERT/+^; Rosa^DTA/+^* mutants, we observed a 58.5% reduction in type I HCs in the utricular striola and a corresponding loss of calyceal nerve endings, compared to controls. In contrast, the proportion of OCM+ type II HCs was not reduced; instead, the total number of striolar type II HCs increased by 55.0% in the *Ocm^CreERT/+^; Rosa^DTA/+^* utricles. Because type I HCs were deleted at birth—when the utricle is still immature and nearly 50% of HCs have yet to appear (16)—it is conceivable that new HCs could be generated to replace those deleted. However, using two independent quantification methods, we found no evidence of recovery in type I HC number, but rather a clear compensatory increase in type II HCs (Fig. 2). Although regenerated type II HCs in adult utricles following substantial HC deletion with DTA remain immature and insufficient to restore vestibular functions (28), it is unclear whether the neonatal increase in type II HCs observed in our study mitigates any of the functional deficits. Importantly, both the structural loss of calyceal endings and the functional deficits observed in our mutants persist into adulthood, indicating that these effects extend beyond early postnatal development.

### VsEPs Require Type I HCs in the Striola.

VsEPs are elicited by repeatedly applying transient linear jerk stimuli to the head, which preferentially activate irregular afferents projecting to the striola of the otolith organs (26, 29–31). Other than the sensory epithelium, the integrity of the otoconia in the otolith organs is important for this function (32, 33). In the *Ocm^CreERT/+^; Rosa^DTA/+^* mutant, the otoconia clearance in the striola of the utricle remained intact (*SI Appendix*, Fig. S3), suggesting that the reduction in VsEPs was not due to abnormal otoconia. Moreover, the extent of amplitude reduction in VsEPs (Fig. 4, 45.9% reduction) is correlated with the extent of reduction in striolar OCM+ type I HCs and pure calyces (Figs. 1 and 3, >70% reduction in both). Together, these results provide direct evidence that striolar type I HCs and their afferents are important for eliciting VsEP response to jerk stimuli.

### VOR Requires Type I HCs in Extrastriolar/Peripheral Zones.

The milliseconds response time demonstrated for VOR in rhesus monkeys (34) has prompted the notion that VOR may be a striolar/central zone function, even though evidence suggests that this reflex is mediated by regular afferents, linking this function to extrastriolar/peripheral zones (35–37). Additional studies also indicate that striolar/central zones are not essential for VOR responses to low frequency stimulations (25, 27). The normal VOR observed in the *Ocm^CreERT/+^; Rosa^DTA/+^* mutants, where extrastriolar/peripheral zones remain intact, supports the conclusion that angular VOR is initiated primarily at extrastriolar/peripheral zones. Given the short latency response of VOR, these results further imply that the nonquantal transmission by type I HCs in extrastriolar/peripheral zones is essential for this function.

If angular VOR is primarily mediated by the peripheral zone, then what is the function of type I HCs in the central zone of cristae? Although VOR remained unaffected in the *Ocm^CreERT/+^; Rosa^DTA/+^* mutants (*SI Appendix*, Fig. S4 and Tables S1–S3), these results do not preclude a critical contribution of irregular fibers within central crista zones in mediating the VOR responses to higher-frequency stimuli beyond the range tested here (34), which fall within the physiologically relevant range (38, 39). Furthermore, primate studies show that irregular afferents predominantly project to vestibular-only (VO) neurons in the vestibular nuclei of the brainstem (36, 40). VO neurons, in turn drive vestibulospinal reflex pathways that control posture and balance, as well as ascending pathways to the thalamus/cortex for spatial orientation (41, 42). This organization framework suggests that selective type I HC loss in striolar/central zones should preferentially disrupt irregular afferent-driven vestibulospinal and ascending pathways—impairing postural and perceptual stability—while leaving regular afferent-driven VOR pathways relatively spared. The preferential reduction of head motion at higher frequencies in the deletion mutants at rest, compared to controls, is consistent with a role for the striolar/central zones in postural control (Fig. 7 *A*–*F*). These deficits indicate that striolar and central-zone type I HCs provide the robust high frequency head-motion signals required for postural stability (reviewed in ref. 7).

### Head Stability Requires Striolar/Central Zone Function.

Postnatal head tremor is the most distinctive phenotype of *Ocm^CreERT/+^; Rosa^DTA/+^* mutants, which otherwise show no obvious vestibular deficits. Such an isolated tremor phenotype is rare among vestibular mutants, apart from one model in which striolar/central zones adopt extrastriolar/peripheral zone identity (27). However, in that study, the head tremor could not be attributed specifically to striolar/central zones dysfunction because potential central defects could not be excluded. Here, by selectively ablating only type I HCs and their calyces in the striolar/central zones, we observed a similar head tremor phenotype, demonstrating that head tremor can arise directly from impaired striolar/central zone function. Since the vestibular system continuously integrates sensory signals from the inner ear with ocular and proprioceptive information, as well as cerebellar inputs to distinguish self-initiated from passive motions (7, 8), we propose that the observed head tremor and instability result from a mismatch between abnormal vestibular inputs and intact extravestibular signals required for postural stabilization.

During normal development, high-frequency components of head motion in postnatal pups declined over time, while the power of head motion increased across frequencies (Fig. 6 *A*–*H*). Despite the reduction of sensory input from the loss of type I HCs in striolar/central zones, this maturation process appears to proceed normally (Fig. 6 *G*–*L*), and head tremor was no longer visually apparent in adult *Ocm^CreERT/+^; Rosa^DTA/+^* mutants. We interpret this apparent behavioral recovery as reflecting maturation of other systems involved in maintaining balance—such as the cerebellar and proprioceptive pathways—thereby reducing reliance on striolar/central zone function in adulthood. However, quantitative head motion analyses revealed persistent abnormalities in adult mutant mice; a general hypopower head motion at rest but a hyperpower head motion on a narrow balance beam, compared to controls (Fig. 7). Both abnormalities were most pronounced at higher frequencies, again consistent with the postulated specialized role of striolar/central zones in encoding rapid head-motion dynamics. Together, these adult deficits are consistent with the view that striolar and central-zone type I HCs—and their irregular afferents—provide the temporally precise head-motion signals necessary for stabilizing posture (43, 44).

Interestingly, similar oscillatory head-movement behaviors have been reported in both nonhuman primates and humans with compromised vestibular function. For example, squirrel monkeys exhibit transient head tremor and postural instability after plugging one of the lateral semicircular canals (45). Patients with chronic bilateral vestibular loss also show unusually pronounced head oscillations when additional loads are applied to the head (46, 47). These head tremors are attributed to absence of vestibular input normally required to maintain head stability and ensure movement accuracy during active goal-directed behaviors (48). Our findings further pinpoint the source of this deficit to the loss of type I HC function in striolar/central zones and raise the importance of head oscillations as a clinical diagnosis.

In conclusion, our genetic studies demonstrated the essential role of type I HCs and their calyces in the striolae and central zones of the inner ear in mediating head stability and supporting postural control. These results also lend strong support to the hypothesis that type I HCs and calyces emerged to meet the demands of head stabilization as vertebrates transitioned from water to land.

## Materials and Methods

### Mice.

The *Ocm^CreERT^* knock-in strain was generated as a contract to the Mouse Genome Engineering Core Facility at the University of Nebraska Medical Center (*SI Appendix*, *Supplementary Methods*). All animal experiments used both sexes and were conducted according to NIH animal user guidelines and approved protocol (#1212).

### Administration of Tamoxifen, Tissue Preparation, Immunohistochemistry, and In Situ Hybridization.

Fifty µL of 1 mg/mL tamoxifen solution in corn oil was injected into the stomach of pups at P0 and P1. Tissue preparation, immunostaining, and in situ hybridization were conducted using standard protocols *SI Appendix*, *Supplementary Methods*.

### Quantification of HCs in Whole-Mount Utricle.

HC numbers were quantified in whole-mount utricle samples stained with anti-OCM, anti-SOX2, and Hoechst. Manual quantification was performed using the open-source FIJI software. *SI Appendix*, *Supplementary Methods*.

### Vestibular Function Assessments.

Vestibular function assessments such as balance beam, head tremor, power spectrum analyses, OKR, VOR, and VsEP were conducted using standard protocols. *SI Appendix*, *Supplementary Methods*.

### Statistics.

All data are represented as mean ± SEM and statistical tests are indicated as described. All *P* values are indicated by asterisks: *P < 0.05, **P < 0.01, ***P < 0.001, and ****P < 0.0001.

## Supplementary Material

Appendix 01 (PDF)

Movie S1. Two P10 littermates injected with tamoxifen at P0 and P1. The *Ocm^CreERT/+^; Rosa^DTA/+^* mouse (upper left) shows a head tremor compared to the littermate *Rosa^DTA/+^* control (lower right). (n = 8 controls, 13 mutants)

## Data Availability

The data that support the findings of this study are available at Dryad [https://doi.org/10.5061/dryad.2jm63xt35 (49)]. All other data are included in the manuscript and/or supporting information.
