# Supplementary material for: Type I hair cells of striolar and central zones in vestibular organs are essential for head stability and postural control
Source: Proc Natl Acad Sci U S A. 2026 Jun 1;123(23):e2535179123. doi: 10.1073/pnas.2535179123 (PMC13250592; doi:10.1073/pnas.2535179123)
Supplement: Supplementary file 1 — Appendix 01 (PDF) [file pnas.2535179123.sapp.pdf]

PNAS

Supporting Information for

Type I hair cells of striolar and central zones in vestibular organs are essential for head stability and postural control

[1,#,+]Kazuya Ono, [1,+]Hyun Jae Lee, [2]Hui Ho Vanessa Chang, [2]Brandie Morris Verdone, [3]Talah Wafa, [1,##]Youngrae Ji, [1,###]Austin Huang, [3]Tracy Fitzgerald, [2,*]Kathleen Cullen and [1,*]Doris K. Wu

[1]National Institute on Deafness and Other Communication Disorders, National Institutes of Health, Bethesda, Maryland, 20892, USA

[2]Department of Biomedical Engineering, School of Medicine, Johns Hopkins University, Baltimore, Maryland, 21205, USA

[3]Mouse Auditory Testing Core Facility, National Institute on Deafness and Other Communication Disorders, National Institutes of Health, Bethesda, MD 20892, USA

Division of Glocal Pharmacology, Department of Pharmacology, Graduate School of Medicine, Osaka University, Osaka, Japan

Sensory and motor systems Research Group, Korea Brain Research Institute (KBRI), Daegu, 41068, South Korea

Department of Otolaryngology - Head and Neck Surgery, Institute for Stem Cell Biology and Regenerative Medicine, Stanford University School of Medicine, Stanford, CA 94305, USA.

[+]Co-first authors
*Co-corresponding authors

Doris K. Wu
Email: wud@nidcd.nih.gov

This PDF file includes:

- Supplementary methods
- Figures S1 to S4
- Tables S1 to S3
- Legend for Movie S1
- SI References

Other supporting materials for this manuscript include the following:
Movies S1

Supplementary methods

Generation of *Ocm^CreERT*. A single strand DNA (ssDNA) containing *CreERT2* (https://www.addgene.org/browse/sequence/199783/) and SV40 polyA sequence with 90 bp homology arms were custom synthesized by Integrated DNA Technologies. At the same time, a guide RNA (crRNA) targeting the nearby 1st ATG of the *Ocm* gene (GTGATGCTCATTTTCTACC) was designed. Using Easi-CRISPR technology, ssDNA generated above was inserted into the *Ocm* locus to generate knock-in mice (1, 2). The guide RNA used was GTGATGCTCATTTTCTACC, which cleaves near the start codon of the *Ocm* gene. The $Ocm^{CreERT/+}$ mice were viable and fertile, and maintained in the C57BL/6J background.

Mouse breeding. $Ocm^{CreERT/+}$ males were crossed with either $Gt(ROSA)26Sor^{tm1(DTA)Lky}/J$ ($Rosa^{DTA/DTA}$, JAX# 009669) or $Gt(ROSA)26Sor^{tm9(CAG\text{-}tdTomato)Hze}/J$ ($Rosa^{tdTomato/tdTomato}$, JAX# 007905) females to generate $Ocm^{CreERT/+}$; $Rosa^{DTA/+}$ or $Ocm^{CreERT/+}$; $Rosa^{tdTomato/+}$ mice. Genotyping for the Ocm^{CreERT} allele was performed using PCR for the *cre* allele. $Rosa^{DTA}$ and $Rosa^{tdTomato}$ alleles were genotyped by Transnetyx.

Tamoxifen administration. Tamoxifen was administered into the newborn pups as previously described (3). Briefly, tamoxifen powder (10 mg, Sigma-Aldrich, catalog no. T5648) was first dissolved in 250 µl of ethanol and 750 µl of corn oil was added to generate a stock solution of 10 mg/ml. At the time of administration, a working tamoxifen solution (1 mg/ml) was freshly prepared by diluting the stock tamoxifen solution with

corn oil. Tamoxifen (50 µl/ pup) was injected into stomach of each neonatal pup at P0 and P1.

Tissue preparation*.* Timed pregnant females or postnatal mice were euthanized with CO_2 gas. Hemi-sectioned heads of the embryos or dissected bony labyrinths from postnatal mice were fixed in 4% paraformaldehyde in PBS overnight. Then, fixed samples were cryoprotected with 30% sucrose and stored in -80˚C until processing for cryo-sectioning or whole mount dissections.

Whole-mount immunohistochemistry*.* Utricles and anterior and lateral cristae, or saccules were dissected and incubated with blocking buffer, which includes PBS containing 4% normal donkey serum and 0.2% Triton X-100. Then, specimens were incubated with various primary antibodies diluted with blocking buffer at 4°C overnight. The primary antibodies used were as follows: goat polyclonal anti-oncomodulin (1:300 Santa Cruz Biotech), rabbit polyclonal anti-myosin7a (1:1000, Proteus Bioscience, #25-6790), mouse anti-calretinin (1:500, MilliporeSigma, #MAB1568), and mouse anti-tubulin beta III (1:1000; BioLegend, #801202). After primary antibody incubation and extensive washing, secondary antibody conjugated with fluorescent proteins (anti-IgG H+L antibody, Thermo Fisher Scientific) was applied and incubated for 1 hour and washed. Then, specimens were mounted in ProLong Gold Antifade (Invitrogen) and imaged using a Zeiss LSM780 confocal microscope.

In situ hybridization. In situ hybridization was conducted as described (4). A digoxigenin-labeled 502 bp RNA probe was generated for mouse *Ocm* using forward and reverse primers, cccagacacctttgaaccac and aggccttgtctgccagatg, respectively.

Quantification of HCs in whole-mount utricle. A total of 10 control utricles and 4 mutant utricles were analyzed. Confocal z-stack images of the entire utricle after staining with anti-OCM, anti-SOX2 and Hoechst, were imported into FIJI. Three regions of interest (ROIs, 44 x 44 µm) each from the striola and lateral extrastriola, and two ROIs from the medial extrastriola were selected (Fig. 2A, D). Positions of ROI were selected from defined areas of control utricles based on OCM staining pattern. This map was saved in the FIJI ROI Manager and consistently applied to all samples. ROI locations were manually adjusted due to minor variations in shape and size of each utricle, when necessary.

For cell counting, Hoechst+ HC nuclei in each ROI were identified using the Fiji Cell Counter plugin and marked throughout the entire z-stack images. Supporting cell nuclei were readily distinguishable and excluded, based on their smaller size and their basal locations within the sensory epithelium. Type II HCs were identified and counted based on nuclei that were co-labeled with both Hoechst and SOX2. Total type I HC numbers were calculated by subtracting SOX2+ HC nuclei from the total number of HC nuclei.

Imaging of otoconia. Light microscopy (Leice DFC 7000) was applied for imaging otoconia formation in P8, P16 mouse utricle. Tamoxifen was administered into the newborn pups at P0 and P1. Temporal bones were collected and fixed with 4% PFA in

PBS overnight. After wash with ice-cold PBS three times, utricle samples were dissected and imaged on a glass-bottom dish.

Vestibular function assessments

Balance Beam Assessments*.* Mice traversed a balance beam approximately 70 cm above the ground. The beam was 6 mm wide and 80 cm long. Animals were placed at the midpoint and traversed 40 cm towards a goal box (approximately 10 x 7 x 6 cm, entrance diameter approximately 3.5 cm) with an inertial measurement unit (IMU) affixed to the head as previously described (5, 6). Time to cross was calculated as the time the mouse was placed at the midway point to the time the mouse entered the goal box. Mice were scored as “time out” if they failed to reach the goal within 2 minutes. Significance was calculated using an unpaired, two-tailed t-test. While performing the task, six dimensions of head movements were recorded as previously described (5). Data was acquired at 200 Hz for subsequent spectral analysis (see Power Spectrum Analysis below).

Power Spectrum Analysis. For all instrumented assessments using an IMU, data were up-sampled to 1 kHz for analysis. Power spectral densities (PSD) were computed using Welch’s averaged periodogram (pwelch function, MATLAB, MathWorks) with number (length) of fast fourier transform = 4096 and a Hamming window (4,096 ms duration) for all six axes of movement. These axes include three linear translational axes (forward and backward [foreaft], left and right [lateral] and up and down [vertical]) and three rotational axes (roll, pitch and yaw). To calculate significance, a non-parametric permutation test using a moving-frequency window approach was implemented. PSD

values were averaged using a moving window at each frequency bin across the 0.1–30 Hz range. For each window, a non-parametric permutation test with 1000 repetitions was performed. This procedure was repeated independently for each sensor axis. Significance values were visualized using color-coded shading with thresholds.

***Tremor Assessments**.* For tremor testing at P8, pups were placed in a cylindrical area (9 cm diameter and 21.5 cm height) on a heating pad with an IMU affixed to the head using a skin-safe, water-soluble adhesive. Motor activity was recorded for 1 minute in triplicate with overhead video recording. For adult tremor testing (aged approximately 7–8 months), the IMU was affixed to the head in the same manner as during balance beam assessments. Mice were placed in the cylindrical arena and motor activity was recorded for 2 minutes.

***Vestibulo-ocular reflex (VOR) and Optokinetic reflex (OKR) Assessments**.* Mice underwent surgical and experimental procedures as previously described (7). Real-time eye position was recorded using an infrared video-based tracking system (ETL-200, ISCAN Inc.). Head-on-body velocity was measured with a MEMS inertial sensor (MPU-9250, SparkFun Electronics) mounted on a turntable to capture rotational velocity. Optokinetic reflex (OKR) responses were elicited by sinusoidal rotation of a visual surround composed of vertical black and white stripes (5° visual angle width) while the mouse remained stationary at the center of the turntable. The surround was rotated at frequencies of 0.2, 0.4, 0.8, 1, and 2 Hz with peak velocities of ± 16°/s. Vestibulo-ocular reflex (VOR) responses were recorded by rotating the turntable at the same frequencies

and velocities. For VOR in the light, the visual surround remained stationary; in the dark condition, both the turntable and surround rotated in phase to eliminate visual cues. Head and eye movement signals were low-pass filtered at 125 Hz and digitized at 1 kHz. Velocity was acquired by differentiating position signals, and segments containing eye movement quick-phases were excluded from analysis. VOR and OKR gain and phase were calculated using least-squares optimization, and results were expressed as mean ± standard error of the mean (SEM), across animals at each stimulus frequency. Significance was calculated using two-way ANOVA, with Sidak's multiple comparisons test.

VsEP measurements. VsEPs were recorded in *Rosa*$^{DTA/+}$ and *Ocm*$^{CreERT/+}$*; Rosa*$^{DTA/+}$ mice. VsEPs recordings were based on published methods (8). Briefly, mice were placed supine on a heating pad held at 37°C to maintain body temperature. Subcutaneous electrodes were placed at the nuchal crest (noninverting), the left ear (inverting) and the right hip (ground). The head with the nose pointing upward was placed in a noninvasive head clip lined with weather stripping.

A mechanical shaker (Labworks Inc. ET 132-2, Costa Mesa, CA, USA) coupled to a head clip via a custom metal plate was used to deliver the stimuli. Stimuli were linear acceleration ramps (2-ms (millisecond) duration) delivered at a rate of 17/sec to a power amplifier (QSC Rmx 850, Costa Mesa, CA, USA) which drove the mechanical shaker. The resultant translational head motion was in the naso-occipital axis, stimulating both the saccule and utricle of the mouse.

The stimulus was calibrated and monitored throughout testing in jerk, the first derivative of acceleration (da/dt). The output of a calibrated accelerometer (VibraMetrics Model

1018, Princeton Junction, NJ, USA) mounted on the same custom plate to which the head clip was attached was routed to a custom-built differentiator to convert it to jerk in units of g/ms (where 1g = 9.8 m/s^2; (9, 10)). Stimulus level was measured as the mean peak jerk amplitude and was expressed in dB (re: 1 g/ms).

Figures

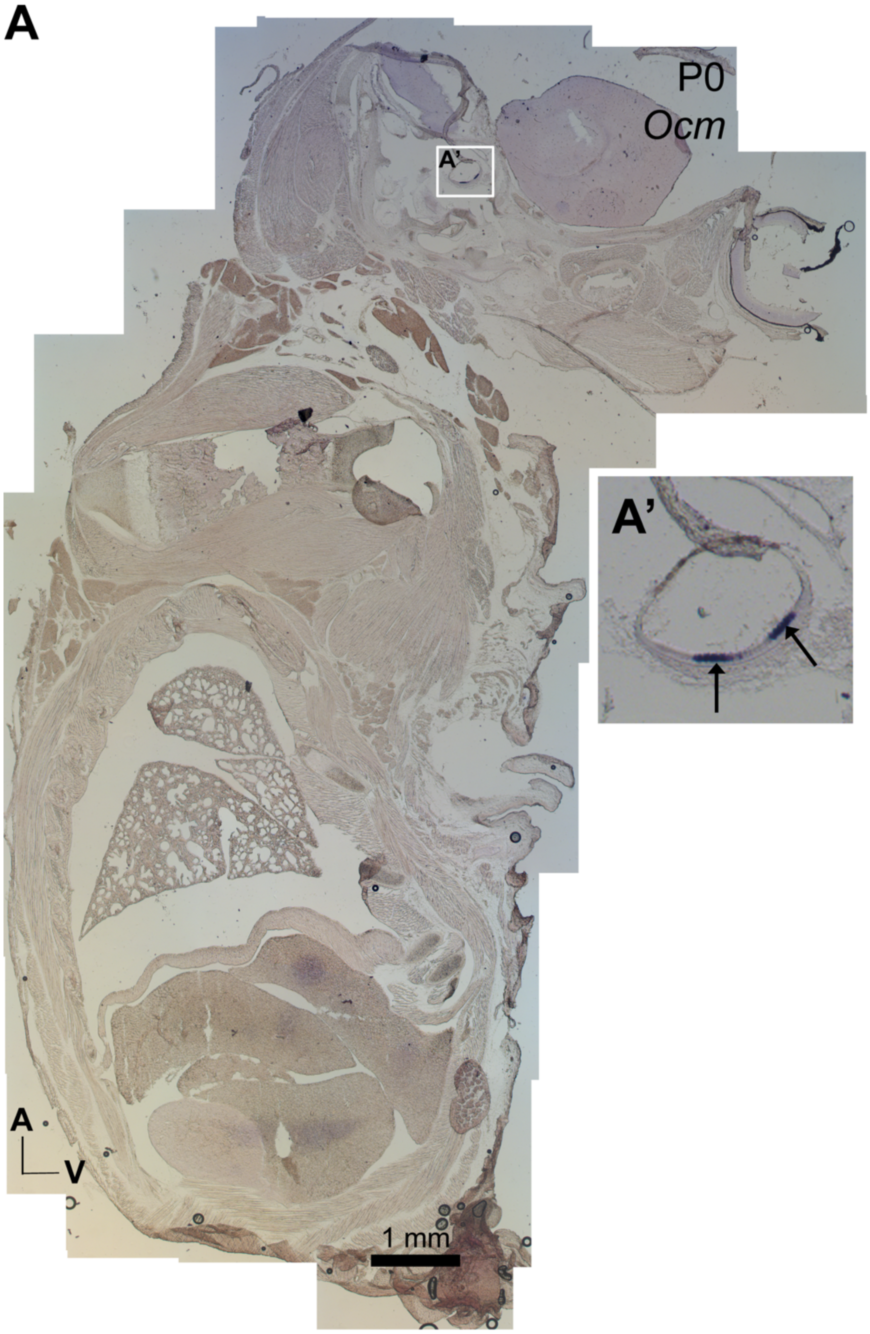

Fig. S1. Expression of *Ocm* in P0 mouse. (A, A') Sagittal section of neonatal mouse including brain, inner ear, and internal organs, hybridized with antisense *Ocm* RNA probe. Only the inner ear shows clear *Ocm* expression (arrows in A'). A, anterior; V, ventral. Scale bar: 1 mm.

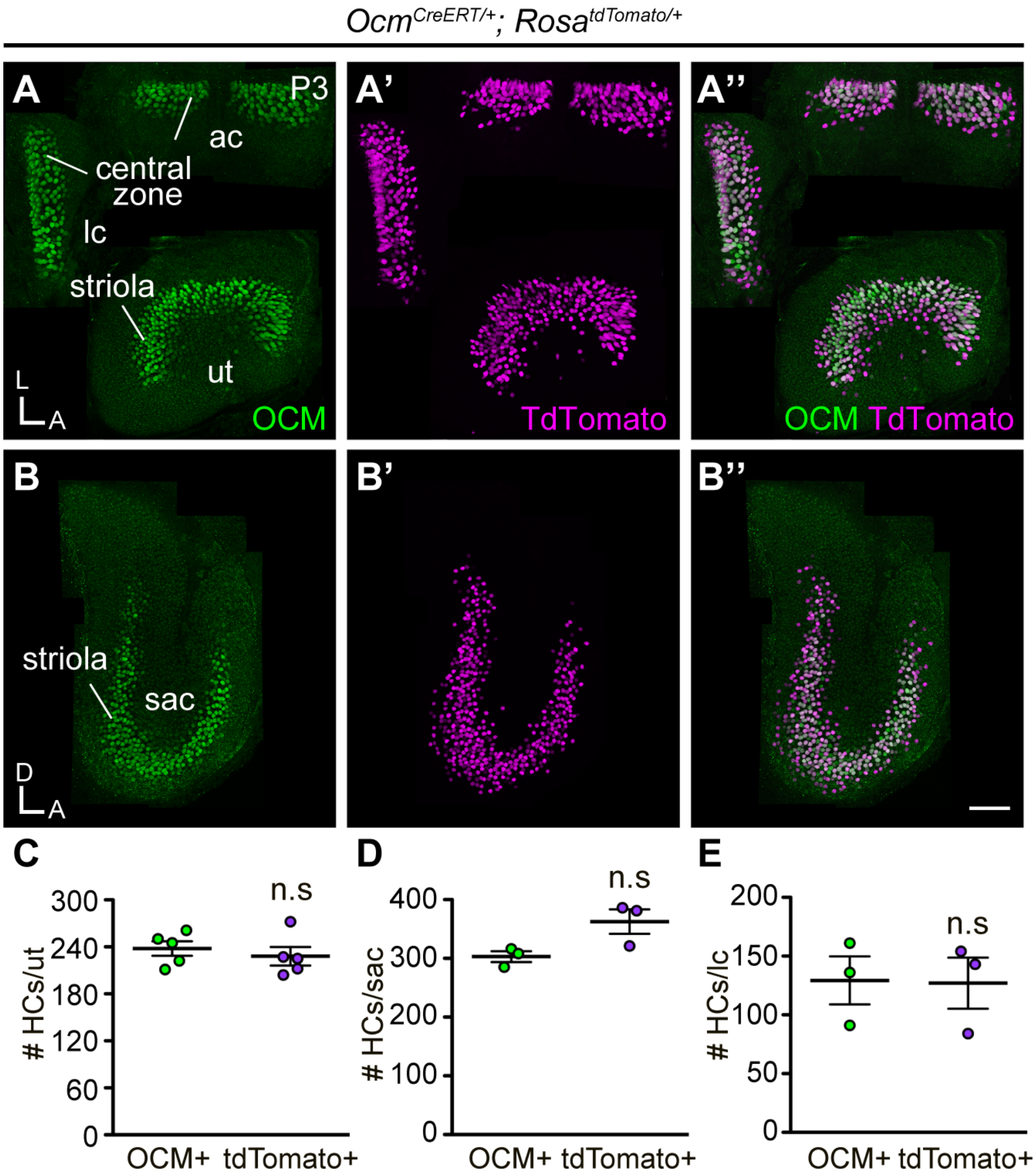

Fig. S2. *Ocm^{CreERT}* reporter activity in the vestibular organs. (A-B) Whole mount preparations of *Ocm$^{CreERT/+}$*; *Rosa$^{tdTomato/+}$* sensory organs of (A) anterior crista, lateral crista and utricle and (B) saccule immunolabeled with anti-oncomodulin (OCM, green) antibody. TdTomato expression (magenta) after tamoxifen administration at P0 and P1 shows a strong overlap with OCM^{+} HCs in the striola of the utricle (ut, A-A'') and saccule (sac, B-B''), as well as central zone of anterior (ac, A-A'') and lateral cristae (lc, A-A'') by P3. Most OCM^{+} HCs are co-labeled with tdTomato in all each sensory organ. (C-E) No significant difference in the number of OCM^{+} and the number of tdTomato^{+} HCs in utricles (C, 237.8 ± 9.3 vs. 228.0 ± 11.8, n = 5, p = 0.5311, unpaired t-test), saccules (D, 303.0 ± 9.3 vs. 362.7 ± 20.1, n = 3, p = 0.0593) and lateral cristae (E,129.3 ± 20.5 vs. 127.0

± 21.3, n = 3, $p = 0.9414$) were observed. Error bars: SEM. A, anterior; L, lateral; D, dorsal. Scale bar in B” equals 200 μm and applies to A-B’.

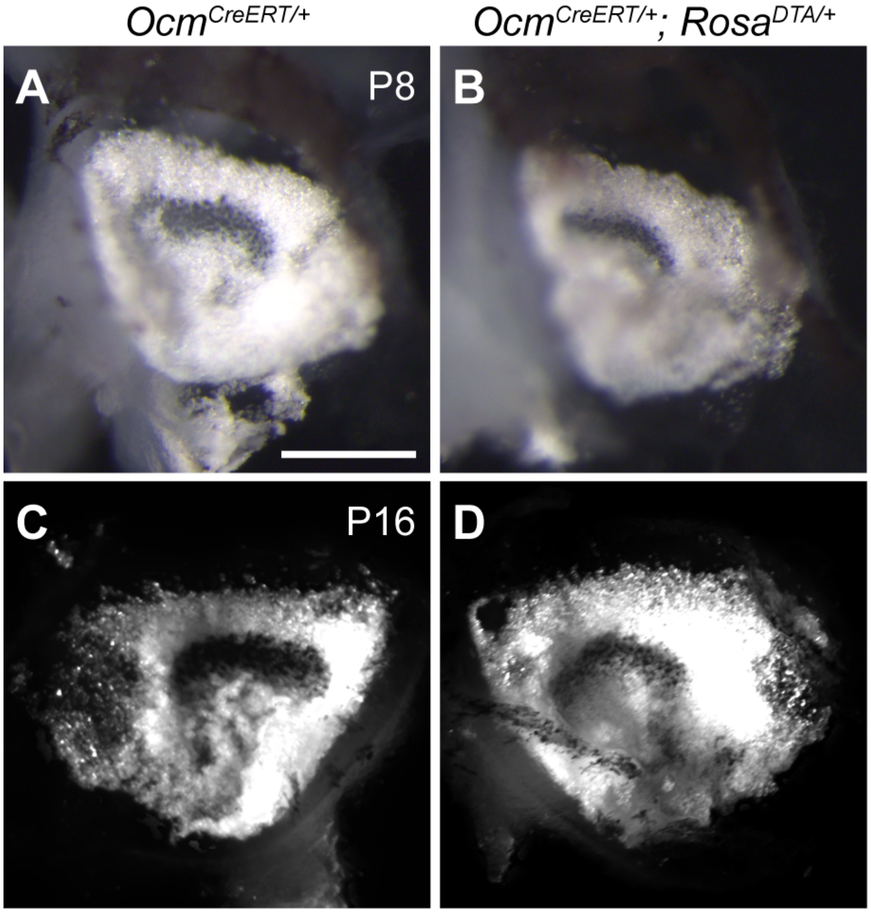

Fig. S3. Similar otoconia clearance associated with the striola of the utricle between controls and mutants. Representative light microscopic images of control (A, C) and *Ocm*$^{CreERT/+}$; *Rosa*$^{DTA/+}$ mutant (B, D) utricles at P8 (A, B) and P16 (C, D). Otoconia is the white crystalline layer on top of the sensory epithelium and appears transparent above the striola due to the smaller and thinner crystals. In both P8 (n = 1) and P16 (n = 2), mutant utricles exhibited a similar level of otoconia transparency above the striola, compared to controls. Scale bar: 200 μm.

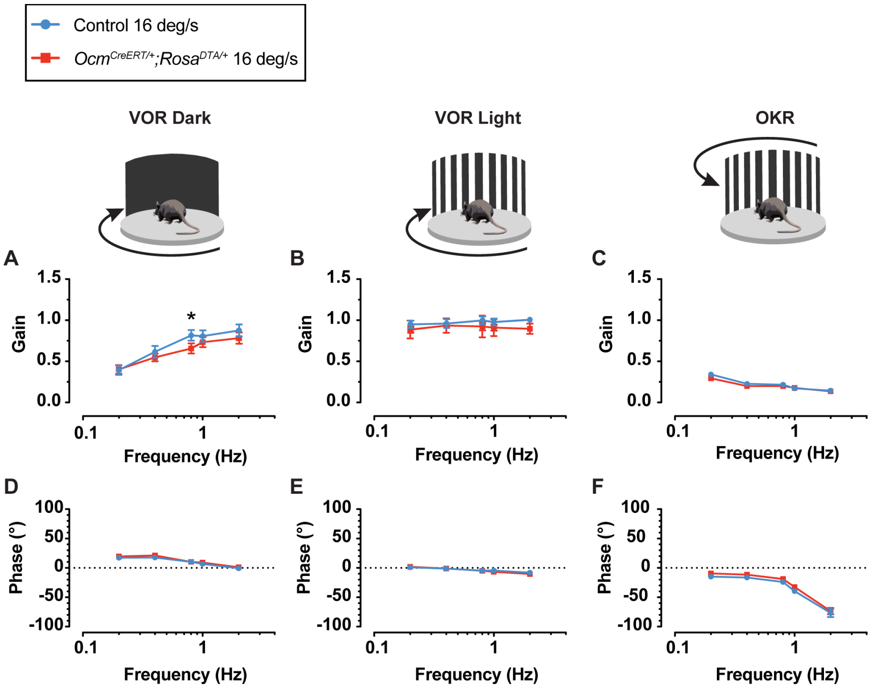

Fig. S4. VOR and OKR assessments are similar between controls and *$Ocm^{CreERT/+}$; $Rosa^{DTA/+}$* mutants. (A-C) Comparison of gains from control and *$Ocm^{CreERT/+}$; $Rosa^{DTA/+}$* mice in (A) VOR in dark conditions, (B) VOR in light conditions, and (C) OKR conditions. n = 8 control and 8 *$Ocm^{CreERT/+}$; $Rosa^{DTA/+}$* mice. Means ± SEM. Two-way ANOVA, Sidak's multiple comparison's test. (A) VOR in dark conditions: Frequency x Gain p = 0.2884. Control and *$Ocm^{CreERT/+}$; $Rosa^{DTA/+}$* demonstrated a significant difference in gain at 0.8 Hz (p = 0.0222, asterisk). (B) VOR in light conditions: Frequency x Gain p = 0.8785. (C) OKR: Frequency x Gain p = 0.6933. (D-F) Comparison of phase in degrees from control and *$Ocm^{CreERT/+}$; $Rosa^{DTA/+}$* mice in (D) VOR in dark conditions. (E) VOR in light conditions, and (F) OKR conditions. Means ± SEM. Two-way ANOVA, Sidak's multiple comparison's test. (D) VOR in dark conditions: Frequency x Phase p = 0.9687. (E) VOR in light conditions: Frequency x Phase p = 0.1182. (F) OKR: Frequency x Phase p = 0.9894. Summary statistics included in Tables S1-3.

Table S1. Comparison of gain and phase from control and *Ocm*$^{CreERT/+}$; *Rosa*$^{DTA/+}$ mice during VOR in dark conditions. Related to Figure S4A, D.

VOR DARK	Gain			Phase (°)		
**Frequency (Hz)**	**Control**	**Mutant**	**P-Value**	**Control**	**Mutant**	**P-Value**
0.2	0.39 ± 0.057	0.40 ± 0.054	>0.99	17.17 ± 3.22	19.62 ± 3.14	0.9603
0.4	0.61 ± 0.070	0.55 ± 0.048	0.6756	17.71 ± 2.09	21.12 ± 1.98	0.8578
0.8	0.81 ± 0.064	0.66 ± 0.061	0.0222	10.22 ± 2.59	10.26 ± 1.53	>0.9999
1.0	0.81± 0.070	0.73 ± 0.061	0.6050	6.80 ± 2.40	9.14 ± 2.14	0.9672
2.0	0.87 ± 0.073	0.78 ± 0.068	0.3403	-0.72 ± 1.84	1.08 ± 2.99	0.9897

Table S2. Comparison of gain and phase from control and *Ocm*$^{CreERT/+}$; *Rosa*$^{DTA/+}$ mice during VOR in light conditions. Related to Figure S4B, E.

VOR LIGHT	Gain			Phase (°)		
Frequency (Hz)	Control	Mutant	P-Value	Control	Mutant	P-Value
0.2	0.95 ± 0.043	0.89 ± 0.039	0.8082	0.95 ± 0.47	1.85 ± 0.38	0.9012
0.4	0.96 ± 0.051	0.94 ± 0.031	0.9968	-1.19 ± 0.56	-1.08 ± 0.34	>0.9999
0.8	1.0 ± 0.046	0.93 ± 0.047	0.6861	-4.46 ± 0.51	-4.86 ± 0.43	0.9971
1.0	0.98 ± 0.040	0.91 ± 0.036	0.7398	-4.46 ± 1.27	-6.57 ± 0.51	0.1776
2.0	1.0 ± 0.029	0.90 ± 0.023	0.2686	-8.01 ± 2.13	-10.26 ± 1.20	0.1332

Table S3. Comparison of gain and phase from control and *Ocm*$^{CreERT/+}$; *Rosa*$^{DTA/+}$ mice during OKR. Related to Figure S4C, F.

OKR	Gain			Phase (°)		
Frequency (Hz)	Control	Mutant	P-Value	Control	Mutant	P-Value
0.2	0.34 ± 0.017	0.29 ± 0.027	0.3649	-14.74 ± 1.28	-9.35 ± 1.43	0.8107
0.4	0.96 ± 0.051	0.94 ± 0.031	0.8204	-16.35 ± 2.14	-11.54 ± 1.89	0.8725
0.8	1.0 ± 0.046	0.93 ± 0.047	0.9766	-23.94 ± 2.14	-18.94 ± 1.53	0.8530
1.0	0.98 ± 0.040	0.91 ± 0.036	0.9999	-39.51 ± 3.43	-32.58 ± 3.92	0.6057
2.0	1.0 ± 0.029	0.90 ± 0.023	0.9989	-76.68 ± 7.06	-73.53 ± 5.44	0.9766

Movie S1 (separate file). Two P10 littermates injected with tamoxifen at P0 and P1. The *Ocm*$^{CreERT/+}$*; Rosa*$^{DTA/+}$ mouse (upper left) shows a head tremor compared to the littermate *Rosa*$^{DTA/+}$ control (lower right). (n = 8 controls, 13 mutants)

SI References

1. H. Miura, R. M. Quadros, C. B. Gurumurthy, M. Ohtsuka, Easi-CRISPR for creating knock-in and conditional knockout mouse models using long ssDNA donors. *Nat Protoc* **13**, 195-215 (2018).
2. R. M. Quadros *et al.*, Easi-CRISPR: a robust method for one-step generation of mice carrying conditional and insertion alleles using long ssDNA donors and CRISPR ribonucleoproteins. *Genome Biol* **18**, 92 (2017).
3. M. E. Pitulescu, I. Schmidt, R. Benedito, R. H. Adams, Inducible gene targeting in the neonatal vasculature and analysis of retinal angiogenesis in mice. *Nat Protoc* **5**, 1518-1534 (2010).
4. H. Morsli, D. Choo, A. Ryan, R. Johnson, D. K. Wu, Development of the Mouse Inner Ear and Origin of Its Sensory Organs. *J. Neurosci.* **18**, 3327-3335 (1998).
5. H. H. V. Chang, B. J. Morley, K. E. Cullen, Loss of alpha-9 Nicotinic Acetylcholine Receptor Subunit Predominantly Results in Impaired Postural Stability Rather Than Gaze Stability. *Front Cell Neurosci* **15**, 799752 (2021).
6. N. C. Hughes, D. C. Roberts, B. Tarchini, K. E. Cullen, Instrumented swim test for quantifying motor impairment in rodents. *Sci Rep* **14**, 29270 (2024).
7. M. Beraneck, K. E. Cullen, Activity of vestibular nuclei neurons during vestibular and optokinetic stimulation in the alert mouse. *J Neurophysiol* **98**, 1549-1565 (2007).
8. S. Vijayakumar *et al.*, Vestibular dysfunction, altered macular structure and trait localization in A/J inbred mice. *Mamm Genome* **26**, 154-172 (2015).
9. T. A. Jones *et al.*, The adequate stimulus for mammalian linear vestibular evoked potentials (VsEPs). *Hear Res* **280**, 133-140 (2011).
10. T. A. Jones, S. M. Jones, Short latency compound action potentials from mammalian gravity receptor organs. *Hear Res* **136**, 75-85 (1999).
